# One or two trainees per workplace in a structured multimodality training curriculum for laparoscopic surgery? Study protocol for a randomized controlled trial – DRKS00004675

**DOI:** 10.1186/1745-6215-15-137

**Published:** 2014-04-23

**Authors:** Felix Nickel, Felix Jede, Andreas Minassian, Matthias Gondan, Jonathan D Hendrie, Tobias Gehrig, Georg R Linke, Martina Kadmon, Lars Fischer, Beat P Müller-Stich

**Affiliations:** 1 Department of General, Visceral, and Transplantation Surgery, University Hospital of Heidelberg, Im Neuenheimer Feld 110, 69120 Heidelberg, Germany; 2 Department of Psychology, University of Copenhagen, Oster Farimagsgade 2A, 1353 Copenhagen, Denmark

**Keywords:** Cholecystectomy, Education, Laparoscopy, Minimally invasive surgery, Training

## Abstract

**Background:**

Laparoscopy training courses have been established in many centers worldwide to ensure adequate skill learning before performing operations on patients. Different training modalities and their combinations have been compared regarding training effects. Multimodality training combines different approaches for optimal training outcome. However, no standards currently exist for the number of trainees assigned per workplace.

**Methods:**

This is a monocentric, open, three-arm randomized controlled trial. The participants are laparoscopically-naive medical students from Heidelberg University. After a standardized introduction to laparoscopic cholecystectomy (LC) with online learning modules, the participants perform a baseline test for basic skills and LC performance on a virtual reality (VR) trainer. A total of 100 students will be randomized into three study arms, in a 2:2:1 ratio. The intervention groups participate individually (Group 1) or in pairs (Group 2) in a standardized and structured multimodality training curriculum. Basic skills are trained on the box and VR trainers. Procedural skills and LC modules are trained on the VR trainer. The control group (Group C) does not receive training between tests. A post-test is performed to reassess basic skills and LC performance on the VR trainer. The performance of a cadaveric porcine LC is then measured as the primary outcome using standardized and validated ratings by blinded experts with the Objective Structured Assessment of Technical Skills. The Global Operative Assessment of Laparoscopic Surgical skills score and the time taken for completion are used as secondary outcome measures as well as the improvement of skills and VR LC performance between baseline and post-test. Cognitive tests and questionnaires are used to identify individual factors that might exert influence on training outcome.

**Discussion:**

This study aims to assess whether workplaces in laparoscopy training courses for beginners should be used by one trainee or two trainees simultaneously, by measuring the impact on operative performance and learning curves. Possible factors of influence, such as the role of observing the training partner, exchange of thoughts, active reflection, model learning, motivation, pauses, and sympathy will be explored in the data analysis. This study will help optimize the efficiency of laparoscopy training courses.

**Trial registration number:**

DRKS00004675

## Background

Minimally invasive surgery (MIS) has been established as a standard for many operations in abdominal surgery. However, in comparison to open surgery, laparoscopic surgeons face technical challenges and increased psychomotor demands resulting in an additional learning curve and prolonged operative times [1-4]. Different training modalities enable prospective surgeons to acquire the psychomotor abilities and surgical skills necessary before applying them to patients [5-9]. Current training modalities include box and pelvi trainers with real surgical instruments, organ models, cadavers, cadaveric organs, live animal models, and computer simulators. Cadavers and animal models provide the most realistic training for operations but are of limited availability [10,11]. Box and pelvi trainers enable the acquisition of basic skills with real instruments and are essential for the training of knot tying and suturing. Virtual reality (VR) trainers enable repetitive training of both basic skills and operations in a virtual environment. In addition, trainees receive automated instructions and feedback, and their performance can be recorded in order to monitor training progress [12,13]. However, at their current level of performance, VR trainers still lack realism in terms of tissue interaction and haptic feedback, and performance feedback is often limited to metric parameters such as the instrument path length [11].

Positive learning effects of both box and VR trainers have been shown for practical laparoscopic skills without clear superiority of one over the other. Other studies have proven positive effects on the duration of operations and on the clinical outcome of both training modalities [5-7,14,15]. When box trainers are augmented with cadaveric organs, surgical interventions can be realistically simulated, e.g., laparoscopic cholecystectomy (LC) [16-18]. Online learning platforms provide videos of operations, explanations, and teaching of surgical techniques, the relevant anatomy, and perioperative management [19,20]. The efficacy of online learning modules has been studied with positive results for online learning both alone and in combination with other training modalities [21]. Multimodality training combines the available training modalities for optimal outcome [11,22,23].

The present study is primarily directed at identifying how many trainees should be assigned per workplace in laparoscopy training courses. One may surmise that the option of having one trainee per workplace would be the optimal training environment and lead to the best training outcome. However, there is currently no evidence available for surgical training [24]. Different factors might exert influence on the outcome of training in pairs or alone. Pairs have many potential advantages, such as the exchange of knowledge, technical discussions between training partners, more pauses with active reflection, analysis of errors, and model learning when the partner trains [25-27]. Evidence from training psychology suggests higher efficiency with pauses and feedback between repetitions of exercises, thus supporting the concept “train less and learn more” [28]. For example, positive effects of intraoperative breaks during long operations have been shown to result in lower stress levels while preserving operation time [29]. Observing peers during learning of practical skills in medical education has been shown to accelerate the learning process, e.g., for physical examination skills [30]. On the other hand, the available literature suggests a learning curve with a certain number of repetitions required for reaching proficiency levels of given tasks and procedures in laparoscopic surgery, thereby suggesting that trainees with a workplace to themselves would benefit [24,31-34]. The secondary objective of this study is to analyze individual and general factors that influence laparoscopic learning curves and test results. The factors assessed include sex, cognitive measures, gaming experience, and personal characteristics [35,36].

## Methods

### Objectives

**Figure 1:**
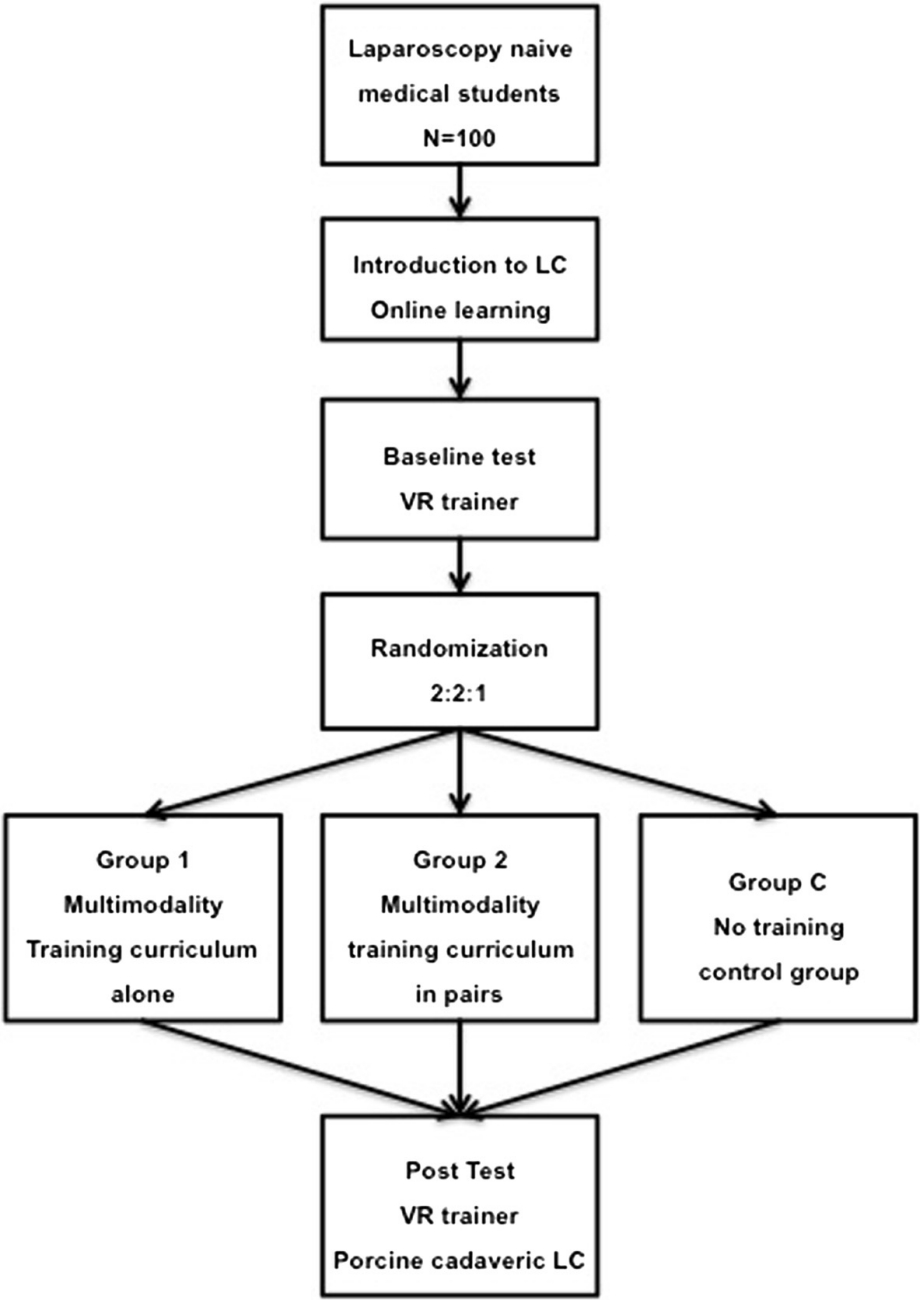
Study protocol flow chart.

The primary goal of this study is to examine whether study participants who undergo laparoscopic training individually (group 1) perform better after the training than study participants who undergo the same training in paired teams (group 2). The control group (group C), which does not receive training, exists to assess the importance of training for a successful operative outcome. Baseline and post-tests are performed on the VR trainer (Figure 1). The operative performance in all three groups will be tested on a cadaveric porcine model with the pulsating organ perfusion (POP) trainer at the end of the study and will be evaluated according to standardized and validated assessment criteria by blinded raters [37-40]. Secondary goals include assessing the influence of individual trainee characteristics on surgical training and performance [35,41,42].

### Study design

This is a registered prospective, single-center, rater-blinded, three-arm, parallel-group randomized controlled trial (DRKS00004675).

### Setting and participants

This study is carried out in the MIS training center of the Department of General, Visceral, and Transplantation Surgery at Heidelberg University Hospital. This study offers voluntary laparoscopic training courses to medical students at Heidelberg University during their clinical years.

### Inclusion and exclusion criteria

Inclusion criteria are students enrolled at Heidelberg University Medical School during their clinical years. Exclusion criteria are students who have already participated in laparoscopy training courses or who have experience assisting in laparoscopic surgeries.

### Introduction to laparoscopic cholecystectomy by online learning

All participants work with online learning modules for two hours as an introduction to LC at the beginning of the study [43]. This is done in a standardized fashion by using the same room at the Department of Surgery at Heidelberg University with identical surrounding conditions in order to rule out any difference between participants. The trainees get assistance with the necessary online registration and are given an explanatory introduction by trained staff in a standardized way to begin the LC module on http://www.webop.de. During this, the trainees are asked to study the anatomy, illustrations, and videos of the procedural techniques. Following this general overview, the participants learn more specific LC information including the operating room set-up and trocar placement. Next, the trainees watch the “*Laparoscopic cholecystectomy: a gold standard case for dissection of Calot’s triangle*” module on http://www.websurg.com to complement what they had previously learned. At the end of the online learning session a standardized multiple choice test is used to check the learning success. The trainees are informed about the multiple choice test at the beginning of the online learning module to ensure that learning motivation is high.

### Introduction to the training modalities in the training center

The participants receive a standardized introduction and instructions on using the box and VR trainer by trained staff. Thus, students can familiarize themselves with the training facilities and training devices prior to the start of the tests and exercises.

### Baseline test

After online learning and a VR introduction, all participants take a baseline test at the VR trainer for an initial assessment of basic skills and LC performance. After this test, participants are randomly assigned to one of the three groups.

### Randomization

Study participants are stratified according to sex and randomly assigned to the training groups or control group in a 2:2:1 ratio by block randomization with a variable block length. Group 1 trains alone, group 2 trains in paired teams, and the control group C receives no training between tests (Figure 1). There are indices for differences between males and females in the acquisition of laparoscopic surgical skills [44,45]. After the participants have finished the baseline test on the VR trainer, an employee of the Department of Surgery at Heidelberg University will perform the randomized distribution of subjects using sealed envelopes. The employee responsible for the randomization and group assignment is otherwise not involved with the training, tests, and data from the present study.

### Training curriculum

The curriculum uses multiple training modalities to ascertain several advantages of each, and to give variety to the trainees for ensuring high motivation. In the present study, the multimodality training embraces online learning, box trainers, and VR training. The training groups participate in a standardized and structured multimodality training curriculum involving box and VR trainers either individually (group 1) or in pairs (group 2). Basic skills are trained with the box (Table 1) and VR trainers (Table 2). Procedural skills and complete LCs are practiced on the VR trainer (Table 3). Training in pairs involves less repetitions of each training task per individual participant since pairs are given the same total training time per workplace and must take turns every 30 minutes. The basic and procedural skills exercises of the curriculum are repeated by the training groups during two training sessions of 4 hours until training time is over. The remaining 4-hour training session is used to repeat the LC modules on the VR trainer. The VR trainer permanently records data from exercises including time, precision, economy of motion, instrumental distance, and the number of misaligned clips.

**Table 1:** Box-trainer basic skills exercises

**Nr.**	**Exercise**
1	Moving matches from a box passing them to the other instrument
2	Crossing of 6 rubber bands in a device of 6 screws
3	Cutting out a predefined circle on paper
4	Drawing a rubber band in a device consisting of eyelets and hooks
5	Cutting out a predefined triangle located on a rubber glove
6	Leading needle and thread in the correct order through eyelets screwed on a board
7	Closing a 5 cm cut on foam with a running suture
8	Attaching a simple interrupted stitch on foam

**Table 2:** VR trainer basic skills exercises

**Basic skill**	**Exercise**
Camera manipulation	The 0° and a 30° angled camera is used to locate 10 balls and snap photos of them
Eye-hand coordination	Objects of blue or red colour have to be touched with the respectively colored instrument tips
Clip-application	Ducts have to be clipped to stop the leakage
Clipping/grasping	Leaking ducts need to be safely grasped and then clipped
Two-handed maneuvers	Balls have to be grasped from a jelly mass and placed into a bag with both instruments
Circular cutting	A circular form has to be freed from tissue attachments by cutting with scissors while retracting the form
Electrocautery	Highlighted tissue bands have to be dissected applying hook cautery

**Table 3:** VR trainer procedural skills exercises for laparoscopic cholecystectomy

**Procedural skill**	**Exercise**
Clipping and cutting	The gallbladder is already exposed with Hartmann’s pouch retracted laterally by a static tool. The cystic duct and artery have to be clipped and cut. Instructions are given.
Exposure of Calot’s triangle	The gallbladder has to be grasped to expose Calot’s triangle. With correct exposure the students are instructed to clip the cystic duct and artery with the second instrument.
Dissection of cystic duct and artery	The gallbladder’s infundibulum has to be retracted for safe dissection of the highlighted cystic duct and artery to achieve the critical view of safety.
Gallbladder separation	The gallbladder is to be separated from the liver bed with eletrocautery. The line of dissection becomes highlighted with adequate retraction.

### Post-test

The post-test includes the basic skills and LC modules on the VR trainer and a porcine cadaveric LC. The groups 1 and 2 take the VR trainer post-test at the end of the training curriculum and the porcine cadaveric LC test on the POP trainer is taken on a separate day.

### Control group

Group C does not participate in the repetitive training exercises, but is provided the same E-learning, LC introduction, and tests as the training groups. Group C takes the VR baseline test on the first day of the study. The post-test on the VR and POP trainer LC are taken on a separate day.

### Blinded test evaluation

The post-test LC with the POP trainer and cadaveric animal organ is used to evaluate and compare the operative performance of all participants. The POP trainer simulates MIS with perfused cadaveric organs. The artery of a hepato-biliary organ can be catheterized and linked to the frequency and pressure controlled POP trainer pump and perfused by colored water. The perfused liquid flows back into the POP trainer via side arms of the arteries, veins, and parenchymal lesions. The Objective Structured Assessment of Technical Skills (OSATS, range 20 to 100) is used as the primary endpoint of the study (Table 4). The expert raters are blinded to the training status of the participants. The OSATS consists of two evaluative spectra and allows for the evaluation of general laparoscopic surgical skills as well as specific procedural and technical skills for the operation. The unweighted sum of the two scales will be evaluated as the primary endpoint.

**Table 4:** Objective structured assessment of technical skills (OSATS) scores

**OSATS**	**Objective structured assessment of technical skills**	**20–100 points**
**GRS**	**General rating scale**	**6–30 points**
	Respect for tissue	1–5 points each
	Time and movement	
	Use and knowledge of instruments	
	Use of camera-assistance	
	Operational duct and anticipation	
	Need of assistance	
**STS**	**Specific technical skills scale**	**14–70 points**
	Retraction of the gallbladder and exposition of Calot’s triangle	1–5 points each
	Preparation of the cystic duct	
	Clipping and sectioning of the cystic duct	
	Preparation of the cystic artery	
	Preparation of the liver bed	
	Specific knowledge about operational techniques	
	Overall quality of the operation	

With the general rating scale (GRS), the rater evaluates the trainee in the following categories (1 to 5 points each): respect for tissue, time and movement, use of instruments, knowledge of instruments, use of camera-assistance, operational duct and anticipation, and need of assistance in general. The GRS covers a range from 6 (minimum) to 30 points (maximum). The specific technical skills scale (STS) measures the retraction of the gallbladder and exposition of Calot’s triangle, preparation of the cystic duct, clipping and sectioning of the cystic duct, preparation of the cystic artery, preparation of the liver bed, specific knowledge about operational techniques, and the overall quality of the operation [38,46]. In this study, the STS scale is applied in a modified way due to specific circumstances: as the criteria “incision and insertion of the port” and “extraction of the gallbladder” refer to surgeries performed on humans or on living animals and cannot be rated properly on the POP trainer, both are replaced by the criteria “knowledge of surgery specific aspects” and “quality of the operative outcome”. The STS covers a range from 14 (minimum) to 70 points (maximum).

In addition to the OSATS criteria, the rater will use the Global Operative Assessment of Laparoscopic Surgical Skills (GOALS, range 6 to 30) score and the time required to perform the operation as secondary endpoints (Table 5) [40]. The given time to finish the LC is 80 minutes so as to ensure the feasibility of this study. Previous studies have shown that 80 minutes is sufficient to assess the competence of each participant for all major parts of the operation.

**Table 5:** Global operative assessment of laparoscopic skills – GOALS score

**GOALS – Global operative assessment of laparoscopic skills**	**6–30 points**
Depth perception	1–5 points each
Bimanual dexterity	
Efficiency	
Tissue handling	
Autonomy	
Level of difficulty	

### Primary outcome measure

The primary outcome measure is the operative performance of the study participants during the porcine cadaveric LC on the POP trainer based on the standardized and validated OSATS score.

### Secondary endpoints

The GRS and STS scales of the OSATS score will be evaluated separately as secondary endpoints, as well as the GOALS scores, operative times, and improvement from baseline to post-test on the VR trainer. In all groups, data from the practical exercises will be collected continuously for each participant based on the multimodal standardized training curriculum designed by the Department of Surgery at Heidelberg University. Each individual exercise on the box trainer will be recorded with respect to time and error. The VR trainer software allows for the continuous recording of various parameters for every participant. Based on this recorded data, learning curves can be displayed for all participants and their differences can be analyzed between groups.

Psychometric and personal parameters will be collected for each participant using anonymous questionnaires. The questions will relate to prior laparoscopic experience and leisure behavior with regards to physical activity, computer games, music, and personal interests. Other parameters, e.g., personality traits and spatial awareness, will also be recorded. Group 2 will receive an additional question concerning team training. In addition, participants will evaluate the training methods through a questionnaire. Explorative analyses will be performed using the collected data and possible relations to the training results [42,47].

### Statistical analysis

The normal distribution provides a fairly exact approximation of the distribution of the scale-specific scores (Figures 1 and two in [46]), which allows standard parametric tests to be used to compare the mean OSATS scores of the three groups [46]. In the first step, overall training effects will be analyzed by a linear mixed model with the main effects Group (1, 2, C), gender (stratification factor), and training pair as a random factor nested in Group 2. If the group effect is not significant at α = 5% in this gatekeeper analysis, statistical inference will stop concluding that there is no substantial training effect at all. If the test is significant, all possible pairs of interventions will be compared (1 vs. C, 2 vs. C, 2 vs. 1) in a similar linear mixed model with group, gender, and training pair (if applicable). These latter analyses will be performed at α = 5% two-tailed, without correction for multiplicity (closed test procedure).

Statistical analysis will be based on the intention-to-treat population that includes all participants that have been randomized and have attended at least one training session. Multiple imputation will be used for missing endpoint information, with linear regression of OSATS scores by the baseline performance on the VR trainer.

Sensitivity analyses will be made using mixed best (Group C) and worst-case (Groups 1 and 2) imputation for the three treatment groups, and vice versa, as well as for the per protocol set of participants that attended all trainings and have complete primary endpoint information.

### Sample size determination

We plan to examine 40 study participants in the two active arms. This sample size, together with a two-sided α = 0.05, gives 80% power to detect a standardized effect of d = 0.64 with a power of 80%. This effect represents approximately 2.5 points for the general skills scale and approximately 3.5 points for the specific skills scale. As the parameters of the general skills scale range from 1 to 5 and those for the specific skills from 2 to 10, the aforementioned effect would reflect an improvement of exactly one scale unit, which is fairly small. The determination of the sample size for the total scores of general and specific scales can only be estimated, as the correlation between the two scales is unknown. Assuming a positive correlation of *ρ* = 0.5, the standard deviation for the total scores of the scales would be 7.86 for both groups. With the sample size of 40 participants per group and α = 0.05 two-tailed, a difference of 5 points would be detected (for example 3 points for general skills area and 2 points for specific skills area) with a power of 80%. Even smaller differences can be neglected.

In the other analyses, the active study arms are compared with the control group, which consists of students who do not take part in any laparoscopic training. The effect of this control group would be larger than the one of the previous case, thereby allowing for a significantly smaller sample size. In each comparison, a standardized effect of d = 0.79 with 80% power can be measured, which would represent a mean difference of about 6 points on the OSATS scale (for example 3 points for general skills area and 3 points for specific skills area).

### Ethical and legal aspects

All data for the study are recorded anonymously, treated confidentially, and are evaluated by authorized staff for scientific purposes only. Participants’ names are kept separate from all study data and are not used for the study. Each participant is assigned a designated code that is used for the entire study documentation and data collection. The study courses are offered in addition to compulsory university courses. Participation in the study is voluntary and may be ended at any time. There are no foreseeable negative consequences for participants related to participation. The participating staff of the Heidelberg MIS center is experienced in the handling of training devices and in tutoring MIS. The benefits of training for students are numerous: stamina, concentration, and manual adroitness are enhanced and practiced, surgical interest may be stimulated or invigorated, and students are able to begin their first practical laparoscopic experience, which may be used during later work. In the event that a participant’s physical or mental health becomes jeopardized due to participation in the present study, the participant will be dismissed immediately and excluded from the study. Ethical approval was obtained by the local ethics committee at Heidelberg University prior to the beginning of the study (Code S-334/2011). Written informed consent is obtained from each trainee.

## Discussion

This study aims to assess the differences in laparoscopy training courses for laparoscopic beginners by using a single workplace to train one or two trainees simultaneously. Since the study participants of Group 2 work in pairs, they repeat each individual exercise less and essentially work for only half of the total training time. Intuitively, this seems disadvantageous, but there is no evidence to support this conjecture. Educating prospective surgeons on common laparoscopic training programs as groups of two might even lead to more successful performances in comparison to solo training. Additionally, this would lead to an effective optimization of resources, as twice the number of trainees could participate in courses.

Despite the careful avoidance of bias, some possible factors of influence still remain, e.g., the contradiction of the high vigilance and motivation of each study participant and the implicated individual difference. Furthermore, it is not ensured that active pauses during exercises always have the same quality for each participant, even though the staff is encouraged to keep making pauses periodically in standardized intervals. We measure if cooperative learning and other exchange effects between the trainees occurs, but not how or why they occur. The expected clinical performance of the study participants will be assessed by the best available simulation, a cadaveric perfused animal organ. Comparison of the two intervention groups will show whether there is a difference in surgical performance based on training alone or in pairs. Comparison of the intervention groups with the control group will show if there exists any training effect at all. The results of the baseline and post-test of the VR trainer should demonstrate the differences in learning success between the trained groups 1 and 2 versus group C without training. The continuous data recording of the VR trainer and the tests will help understand if there is a difference in learning curves between both training groups and between the partners in group 2, as the same training partner always starts the exercises. The assessment of general and individual parameters of the study participants will help understand the possible factors of influence for successful surgical education. As the study is limited to laparoscopically-naive medical students and the performance of basic skills and LC, the results cannot be directly transferred to more experienced surgeons and other interventions. However, the results of this study will increase knowledge about the optimal training conditions for laparoscopic surgery, i.e., if workplaces in surgical training centers should be used by one or two trainees simultaneously in order to achieve the optimal training outcome.

## Trial status

Recruitment started in October 2012 and is planned to be finished in June 2014.

## Abbreviations

GOALS: Global Operative Assessment of Laparoscopic Skills; GRS: General rating scale; LC: Laparoscopic cholecystectomy; MIS: Minimally invasive surgery; OSATS: Objective Structured Assessment of Technical Skills; POP: Pulsating organ perfusion; STS: Specific technical skills scale; VR: Virtual reality.

## Competing interests

The authors hereby declare that they have no competing interests.

## Authors’ contributions

FN: Conception and design, manuscript writing, critical revision, and final approval of the manuscript. FJ: Conception and design, manuscript writing, and final approval of the manuscript. AM: Conception and design, manuscript writing, and final approval of the manuscript. JH: Conception and design, manuscript writing, and final approval of the manuscript. MG: Planning of the statistical analysis and the sample size calculation, manuscript writing, and final approval of the manuscript. TG: Conception and design, critical revision, and final approval of the manuscript. GL: Conception and design, critical revision, and final approval of the manuscript. MK: Conception and design, critical revision, and final approval of the manuscript. LF: Conception and design, critical revision, and final approval of the manuscript. BM: Conception and design, critical revision, and final approval of the manuscript. All authors read and approved the final manuscript.
